# Inactivation of the Antibacterial and Cytotoxic Properties of Silver Ions by Biologically Relevant Compounds

**DOI:** 10.1371/journal.pone.0094409

**Published:** 2014-04-11

**Authors:** Geraldine Mulley, A. Tobias A. Jenkins, Nicholas R. Waterfield

**Affiliations:** 1 School of Biological Sciences, University of Reading, Reading, United Kingdom; 2 Department of Chemistry, University of Bath, Claverton Down, Bath, United Kingdom; 3 Division of Microbiology and Infection, Warwick Medical School, Warwick University, Coventry, United Kingdom; Queen's University Belfast, United Kingdom

## Abstract

There has been a recent surge in the use of silver as an antimicrobial agent in a wide range of domestic and clinical products, intended to prevent or treat bacterial infections and reduce bacterial colonization of surfaces. It has been reported that the antibacterial and cytotoxic properties of silver are affected by the assay conditions, particularly the type of growth media used *in vitro*. The toxicity of Ag^+^ to bacterial cells is comparable to that of human cells. We demonstrate that biologically relevant compounds such as glutathione, cysteine and human blood components significantly reduce the toxicity of silver ions to clinically relevant pathogenic bacteria and primary human dermal fibroblasts (skin cells). Bacteria are able to grow normally in the presence of silver nitrate at >20-fold the minimum inhibitory concentration (MIC) if Ag^+^ and thiols are added in a 1∶1 ratio because the reaction of Ag^+^ with extracellular thiols prevents silver ions from interacting with cells. Extracellular thiols and human serum also significantly reduce the antimicrobial activity of silver wound dressings Aquacel-Ag (Convatec) and Acticoat (Smith & Nephew) to *Staphylococcus aureus, Pseudomonas aeruginosa* and *Escherichia coli in vitro*. These results have important implications for the deployment of silver as an antimicrobial agent in environments exposed to biological tissue or secretions. Significant amounts of money and effort have been directed at the development of silver-coated medical devices (e.g. dressings, catheters, implants). We believe our findings are essential for the effective design and testing of antimicrobial silver coatings.

## Introduction

In recent years, the emergence and persistence of bacterial strains with resistance to multiple classes of antibiotics has led to renewed interest in the antimicrobial properties of silver. There has been a surge in the number of products on the market, both domestic and clinical, that contain antimicrobial silver compounds or nanoparticles. These include anti-odor fabric coatings, deodorants, washing machine filters, laptop coatings, topical burn creams, wound dressings and medical devices [1]–[3]. The development of improved antimicrobial silver coatings and silver nanoparticles continues to receive significant research funding worldwide [4]–[6]. A key aim of this research is to ensure that silver ions are released at a sufficient rate and concentration to be effective as an antimicrobial at levels that are safe for use. This is particularly important for the development of medical devices, such as wound dressings, catheters, bone implants and cardiovascular stents, which are typically tested first *in vitro* (antimicrobial assays and human cell culture) and later *in vivo* (animal models and clinical trials). Topical silver solutions (0.5% silver nitrate) and creams (1% silver sulfadiazine) have been used in the prevention and treatment of wound infections for several decades, but these preparations need to be reapplied frequently in order to penetrate wound tissues due to rapid complexation of silver with wound exudates [7]. Modern advances in silver delivery methods have seen the introduction of sustained release dressings such as the nanocrystalline wound dressing Acticoat (Smith & Nephew) and the hydrogel dressing Aquacel-Ag (Convatec). These dressings should release sufficient Ag^+^ to prevent or reduce bacterial colonization of the wound bed and support efficient healing. Silver coatings on indwelling medical devices have also been developed, such as the Bardex IC Foley catheter (Bard Medical). These coatings should release sufficient silver to reduce or prevent bacterial attachment and formation of biofilms whilst inducing minimal damage to surrounding human cells and tissue [8]. However, differences in experimental conditions and procedures can make comparisons of antimicrobial efficacy and human toxicity from *in vitro* and *in vivo* experiments difficult [9]–[11]. A recent study by Greulich *et al.* used identical growth conditions for bacteria and human cells and this revealed that the antibacterial and cytotoxic properties of both silver ions (silver acetate) and silver nanoparticles are within the same range [10].

Several studies have shown the antibacterial and cytotoxic properties of silver are affected by the assay conditions, including the type of growth media and growth supplements such as fetal calf serum [12]. Only a few studies have explored the chemistry behind these differences. Liau *et al.* showed that compounds containing thiol groups reduce the toxicity of silver to *Pseudomonas aeruginosa* [13]. Similarly, equimolar concentrations of the thiol containing amino acid cysteine reduce the toxicity of silver to *Staphylococcus epidermidis* [14]. The major blood protein serum albumin reduces both the antimicrobial and cytotoxic properties of silver nanoparticles embedded in hydrogels, although the mechanism of inactivation is not known [15].

Whilst the majority of the thiol groups in the proteins of human cells are in the oxidized state (forming disulphide bridges between cysteine residues in proteins), the thiol groups of bacterial cytoplasmic proteins are mostly in the reduced state due to the redox conditions in the prokaryotic cytoplasm [16]. Animals and bacteria have a thiol based antioxidant system that protects cellular components against oxidative damage from reactive oxygen species (ROS) and free radicals. In humans and many Gram-negative bacteria, such as *Escherichia coli* and *P. aeruginosa,* the system utilizes the tripeptide glutathione as the predominant antioxidant. Glutathione is synthesized by specific enzymes from the amino acids glutamate, glycine and cysteine [17], [18]. Following oxidation by ROS, the oxidized glutathione (GSSG) is recycled back to the reduced form (GSH) by the enzyme glutathione reductase using NADPH as an electron donor. In other bacteria such as *S. aureus* and *Bacillus* spp. that cannot synthesize glutathione, the predominant cellular antioxidant is typically a low molecular weight compound synthesized from cysteine [19], [20].

In this study we present the first detailed analysis of the extent to which biologically relevant compounds such as glutathione, cysteine and human blood components affect toxicity of silver ions to clinically relevant pathogenic bacteria in comparison to human dermal fibroblasts (skin cells). We used the notorious nosocomial opportunistic pathogens *S. aureus* and *P. aeruginosa* in these studies as they are frequently exposed to silver-coated dressings and catheters in clinical settings. Our findings have important implications for the future deployment of silver as an antimicrobial agent in environments exposed to biological tissue or secretions.

## Materials and Methods

### Chemicals and Reagents

Silver nitrate, sodium nitrate, GSH, GSSG, amino acids, human serum albumin and human serum were purchased from Sigma Aldrich and stock solutions were prepared fresh for each assay in sterile Milli-Q water, filter sterilized at 0.22 µm (Millex-GS, Millipore). Propidium iodide and NucBlue (a cell permeable form of Hoechst 33347) were diluted to the recommended working concentration in Dulbecco's phosphate buffered saline (DPBS + calcium, magnesium, glucose and pyruvate), all purchased from Life Technologies.

### Bacterial growth and microbiological assays

*Escherichia coli* K12, *P. aeruginosa* PA01 [21], *S. aureus* MSSA476 and MRSA252 [22] were recovered from frozen (−80°C) glycerol (15% *v/v*) stocks on Luria Bertani (LB) agar plates at 37°C for 24 hr. Single colonies were grown in 10 mL LB broth, 250 rpm, at 37°C for 16–18 hr. Bacteria were then sub-cultured (1∶100) in 10 mL LB broth, 250 rpm, at 37°C for 2–5 hr to exponential phase (OD_600_ 0.4–0.6). Cultures were adjusted to OD_600_  = 0.3 and diluted in LB (1∶50) prior to use in microbiological assays unless otherwise stated.

Stock solutions of chemicals were diluted in sterile Milli-Q water at 50× the concentration desired in the assay. These were then diluted 1∶25 in LB broth, human serum albumin 100 µg/mL dissolved in LB, or 100% human serum where stated. 100 µL of this 2× solution was aliquoted into the appropriate wells of a 96-well flat-bottom transparent plate (Greiner) with 100 µL of bacterial culture prepared as described above (equivalent to ∼5×10^5^ bacteria/well) in technical duplicates, with three biological replicates for each strain. Microplates were incubated in a Fluostar Omega plate reader (BMG) for 24 hr, with continuous orbital shaking at 300 rpm, and absorbance measurements taken at 600 nm every 6 min (20 flashes/well/cycle). The optical density of each individual culture at 16 hr or 24 hr was plotted in OriginPro8 (OriginLab) and Sigmoidal curves fitted using the Boltzman function. Fitted values for each individual curve were used to calculate the mean minimum inhibitory concentration (MIC).

To test the effect of R-SH on the antimicrobial activity of wound dressings, 20 mL molten LB agar (42°C) was inoculated with approximately 1×10^5^ bacterial cells and 200 uL of the appropriate concentration of GSH, mixed well and poured into a standard 90 mm Petri dish. For human serum tests, 2 mL molten LB agar (42°C) was mixed with 2 mL human serum and approximately 2×10^4^ bacterial cells and poured into wells in a 6-well tissue culture dish (Corning). Squares (1.25 cm×1.25 cm) of Aquacel (Convatec), Aquacel-Ag (Convatec) and Acticoat (Smith & Nephew) dressings were applied to the surface of the solidified agar. Plates were incubated for 24 hr at 37°C and the zones of inhibition surrounding the dressings were measured (n = 3). Statistical significance was calculated using Student's *t*-test.

### Human cell culture and cytotoxicity assays

Primary adult human dermal fibroblasts were purchased from the American Type Culture Collection (PCS-201-012). All incubations were at 37°C, 5% CO_2_/95% air in a humidified incubator. Cells were cultured in 75 cm^2^ tissue culture flasks in Medium 106 supplemented with low serum growth supplement (Life Technologies) to a confluence of ∼80% for up to 8 passages. Cells were detached from tissue culture flasks using trypsin-EDTA and trypsin neutralizer solution as per the manufacturer's protocol (Life Technologies).

For cytotoxicity tests, cells were seeded at 5×10^3^ cells/cm^2^ in 24-well dishes with 500 µL media per well and grown to a confluence of ∼80% with media replaced every 24 hr for 2–3 days. Stock solutions of silver nitrate and GSH were diluted 1∶50 in Medium 106 supplemented with low serum growth supplement (NB. the pH of the culture medium was not affected). Plates were incubated for 4 or 24 hr and media was replaced with 500 µL propidium iodide solution and incubated for 20 min. This solution was then replaced with 500 µL NucBlue solution and incubated for 20 min. Micrograph images were captured using an EVOS fl digital inverted microscope (Advanced Microscopy Group) with the light microscope, DAPI light cube (excitation at 357 nm, emission at 447 nm, to detect NucBlue stain) and RFP light cube (excitation at 531 nm, emission at 593 nm, to detect propidium iodide) at ×20 magnification. Stained nuclei were counted in captured images using ImageJ [23] with means and standard errors of the mean calculated from two technical replicate images per well and four biological replicates per condition. The percentages of viable cells (ratio of cells stained with propidium iodide vs. NucBlue) were plotted in OriginPro8 (OriginLab) and Sigmoidal curves fitted using the Boltzman function. Fitted values representing a 50% reduction in viability for each individual curve were used to calculate the mean cytotoxic concentration (CC_50_).

### Quantification of silver

Overnight cultures of *S. aureus* were sub-cultured in 50 mL LB broth in sterile 250 mL Erlenmeyer flasks and grown for 2–3 hr at 37°C with aeration (250 rpm shaking), to OD_600_ 0.5–0.8. 10 mL aliquots of culture were diluted 1∶2 into LB with or without AgNO_3_ and with or without GSH to a final concentration of 1 mmol dm^−3^. Cultures were incubated for 1 hr at 37°C, 250 rpm and cells harvested by centrifugation at 4°C. The supernatant was discarded, cell pellets were washed 3× in 1 mL PBS and re-suspended in 200 µL 70% ethanol. Samples were boiled at 90°C for 1 hr to lyse the cells and dry the pellets. Pellets were weighed, re-suspended in 3 mL nH_2_0 and transferred to digestion tubes. 10.5 mL concentrated hydrochloric acid and 3.5 mL concentrated nitric acid was added to each sample to cold digest overnight. Samples were heated to 140°C for 2.5 hr, allowed to cool, and filtered through Cu impregnated filter papers (prepared by soaking Whatman no. 540 filter paper in 0.1 M copper nitrate and rinsing 3× in nH_2_O). Samples were made to volume in 100 mL volumetric flasks with 0.5 M nitric acid and diluted 1∶2 with nH_2_0 prior to analysis by ICP-OES. Three blank samples were prepared without cell pellets as negative controls to set the detection limit.

## Results

### Antibacterial activity of silver nitrate in different conditions

**Figure 1:**
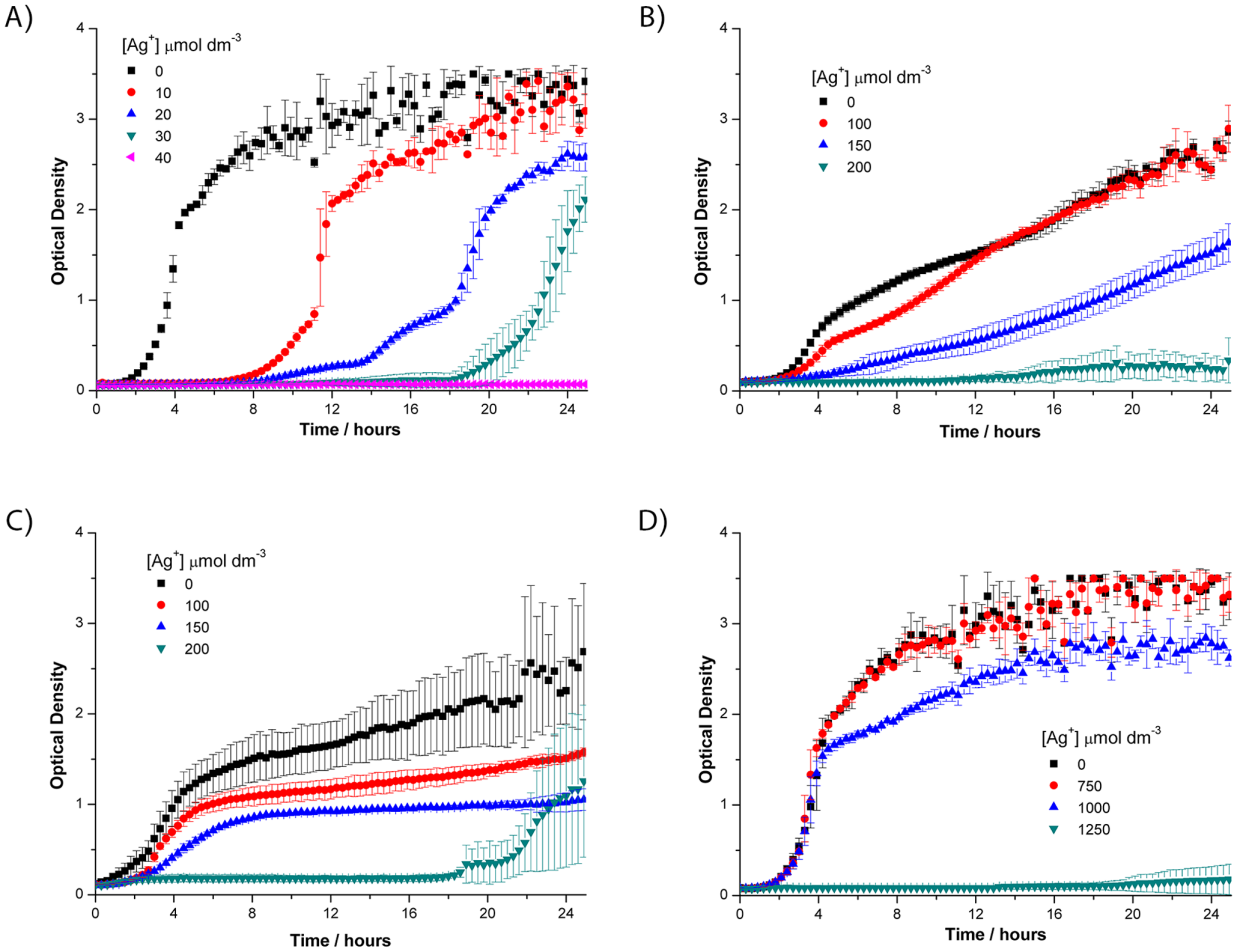
The effect of silver nitrate on the growth of *Staphylococcus aureus* MSSA476 in different media. AgNO_3_ was added to growth media at the indicated concentrations (µmol dm^−3^) in (A) LB; (B) LB + 50 mg/mL HSA; (C) LB + 50% human serum (*v/v*); (D) LB + 1 mmol dm^−3^ GSH. GSH, reduced glutathione; HSA, human serum albumin (the major blood protein). Error bars  =  SD, n = 3.

**Figure 2:**
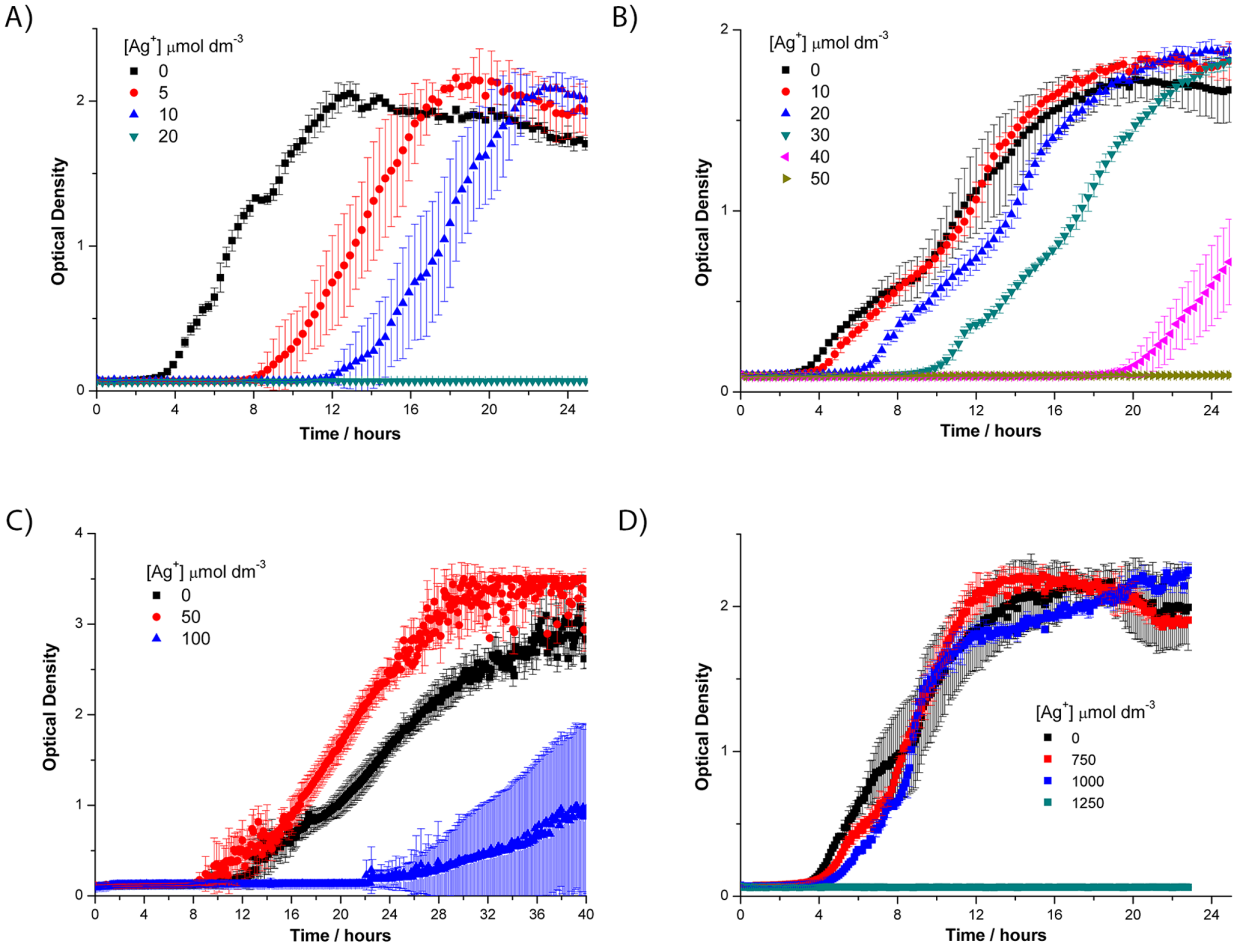
The effect of silver nitrate on the growth of *Pseudomonas aeruginosa* PA01 in different media. AgNO_3_ was added to growth media at the indicated concentrations (µmol dm^−3^) in (A) LB; (B) LB + 50 mg/mL HSA; (C) LB + 50% human serum (*v/v*); (D) LB + 1 mmol dm^−3^ GSH. GSH, reduced glutathione; HSA, human serum albumin (the major blood protein). Error bars  =  SD, n = 3.

The pathogenic clinical isolates *S. aureus* MSSA476 and *P. aeruginosa* PA01 were grown overnight in LB broth with a range of concentrations of silver nitrate, which readily dissolves in culture media to Ag^+^ and NO_3_^−^. Each increase in the concentration of silver nitrate below the minimum inhibitory concentration (MIC) resulted in a prolonged lag phase (i.e. the time between the inoculation of bacteria and the onset of exponential growth) for both strains, but once growth had initiated the growth rate was then comparable to that in LB (Fig. 1A and 2A). The MIC of silver nitrate in LB broth was 33 µmol dm^−3^ to *S. aureus* MSSA476, 13 µmol dm^−3^ to *P. aeruginosa* PA01 and 37 µmol dm^−3^ to *E. coli* K12, at 16 hr (Table 1). A methicillin resistant *S. aureus* strain, MRSA252, was also tested and the MIC was equivalent to that of MSSA476. We found that the MIC was not affected by the number of bacteria in the starting inoculum as 10-fold dilutions of bacteria from 1×10^6^ to 1×10^2^ bacteria per well resulted in comparable MIC values.

**Table 1:** The effect of biologically relevant compounds on the minimum inhibitory concentration (MIC) and cytotoxic concentration (CC_50_) of silver nitrate to bacteria and human cells.

Growth conditions	*P. aeruginosa* µmol dm^−3^	*S. aureus* µmol dm^−3^	*E. coli* µmol dm^−3^	Human Fibroblasts µmol dm^−3^
Media only	13±2	33±3	37±5	23±1
Media + HSA 50 mg/ml	44±2	158±10	50±10	ND
Media + human serum 50% *v/v*	81±7	174±17	ND	ND
Media + 1 mmol dm^−3^ GSH	1126±9	1121±80	1020±65	982±72

The MIC of AgNO_3_ to *P. aeruginosa, S. aureus* and *E. coli* was determined in Luria-Bertani broth and the CC_50_ of AgNO_3_ to primary human dermal fibroblasts in Medium 106 supplemented with low serum growth supplement (± SD, n≥3). GSH, reduced glutathione; HSA, human serum albumin; ND, Not determined (conditions do not support growth).

Coatings on medical devices such as bandages and catheters contact human blood and tissue. To assess whether the components of blood affect the antimicrobial efficacy of silver ions, the MIC of silver was determined in LB supplemented with human serum (blood depleted of cells and clotting factors) and human serum albumin (HSA, the major blood protein present in serum). Both of these blood components increased the MIC of AgNO_3_ to *S. aureus* (Figure 1B and 1C) and *P. aeruginosa* (Figure 2B and 2C), with serum being more potent than HSA alone indicating the presence of additional components within serum that inactivate Ag^+^ toxicity. Note that only 50% serum was used in this experiment so the protective effect of whole blood is potentially greater *in vivo*. The inclusion of 1 mmol dm^−3^ GSH in LB enabled *S. aureus*, *P. aeruginosa* and *E. coli* to grow in the presence of up to, but not in excess of, 1 mmol dm^−3^ AgNO_3_ (Table 1). The lag phase and growth rate in LB with 1 mmol dm^−3^ AgNO_3_ + 1 mmol dm^−3^ GSH was remarkably similar to that in LB with 1 mmol dm^−3^ GSH alone (Figure 1 and 2) indicating GSH causes complete loss of silver ion toxicity in a 1∶1 molar ratio. The addition of 1 mmol dm^−3^ cysteine to LB showed the same protective effect as GSH enabling normal growth up to, but not in excess of, 1 mmol dm^−3^ AgNO_3_ (data not shown). This suggests that silver ions bind to glutathione and cysteine (which both contain one thiol group) in a 1∶1 ratio and that these complexes are not toxic to bacteria. In contrast, the addition of glutamate, glycine, methionine, histidine or cystine (cysteine disulphide) at 1 mmol dm^−3^ did not rescue growth of either *P. aeruginosa* or *S. aureus* at 200 µmol dm^−3^ AgNO_3_ in LB. The addition of 1 mmol dm^−3^ GSSG was toxic (data not shown). We speculate that the addition of excess GSSG would lead to depletion of the cellular pool of reductant as the bacteria attempt to convert it back to GSH. The addition of 1 mmol dm^−3^ sodium nitrate to LB did not affect the growth of the bacterial strains compared to LB alone, indicating NO_3_^−^ does not influence the toxicity of AgNO_3_.

**Figure 3:**
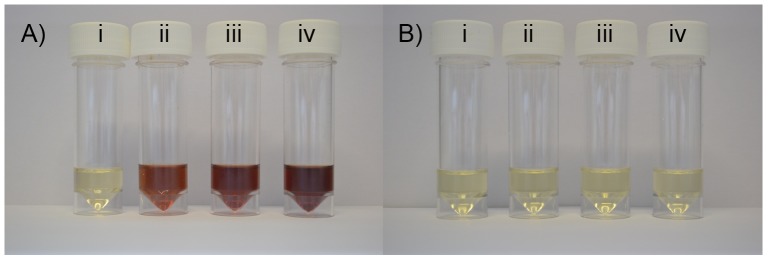
Photochemical reduction of Ag^+^ in LB medium. AgNO_3_ was added to (A) LB and (B) LB + 1 mmol dm^3^ GSH at (i) 0 mmol dm^3^, (ii) 0.75 mmol dm^3^, (iii) 1.0 mmol dm^3^, (iv) 1.25 mmol dm^3^. GSH, reduced glutathione.

After performing these assays, the surplus media was left on the lab bench and we noted that the LB + AgNO_3_ solutions became increasingly dark brown over time, but this was prevented by the addition of 1 mmol dm^−3^ GSH (Figure 3) or cysteine and these solutions remained clear for over 3 months.

### Antibacterial activity of silver-coated dressings in different conditions

Silver coated wound dressings come into contact with biological secretions within the wound bed. The antibacterial properties of wound dressings can be tested *in vitro* by measuring the zone of inhibition surrounding a test sample. Silver ions released from dressings diffuse through the agar and prevent bacterial growth where the concentration exceeds the MIC. Aquacel-Ag (Table 2) and Acticoat (Table 3) dressings showed similar efficacies against the test bacteria in LB agar, with *P. aeruginosa* displaying the largest zone of inhibition as expected based on the greater sensitivity of this species to silver ion toxicity (Table 1). No zones of inhibition were observed for non-silver Aquacel dressings confirming Ag^+^ release is solely responsible for the inhibition of growth caused by Aquacel-Ag and Acticoat. Increasing the concentration of GSH in the LB agar caused a corresponding reduction in the size of the zone of inhibition caused by both Aquacel-Ag and Acticoat. The inclusion of human serum (50% *v/v*) in the agar significantly reduced the size of the zone of inhibition to *P. aeruginosa* and *S. aureus* (Student's *t*-test P<0.001). Our results confirm that the antimicrobial effectiveness of these dressings is significantly reduced by the presence of extracellular R-SH and human serum (Table 2 and 3).

**Table 2:** The effect of biologically relevant compounds on the antimicrobial efficacy of Aquacel-Ag (Convatec) wound dressings.

Growth conditions	*P. aeruginosa* mm	*S. aureus* mm	*E. coli* mm
LB agar only	8.7±0.6	3.0±0.0	4.3±0.6
LB agar + 0.1 mmol dm^−3^ GSH	6.3±0.6 *	2.0±0.0	2.5±0.5
LB agar + 0.5 mmol dm^−3^ GSH	5.2±0.3 *	0.2±0.3 *	0.3±0.3 *
LB agar + 1 mmol dm^−3^ GSH	1.7±0.6 *	0.0±0.0 *	0.0±0.0 *
LB agar + human serum 50% *v/v*	2.3±0.6 *	0.8±0.3 *	ND

The average zone of inhibition (mm) surrounding 1.25×1.25 cm dressing samples applied to bacterial lawns. ± SD, n = 3, * denotes a significant difference from LB agar only control (Student's *t*-test P<0.01). GSH, reduced glutathione; LB, Luria-Bertani; ND, Not determined (conditions do not support growth).

**Table 3:** The effect of biologically relevant compounds on the antimicrobial efficacy of Acticoat (Smith & Nephew) wound dressings.

Growth conditions	*P. aeruginosa* mm	*S. aureus* mm	*E. coli* mm
LB agar only	9.0±1.0	3.0±0.0	4.3±0.6
LB agar + 0.1 mmol dm^−3^ GSH	7.3±0.6	2.2±0.3 *	2.5±0.5
LB agar + 0.5 mmol dm^−3^ GSH	5.8±0.3 *	0.3±0.3 *	0.8±0.3 *
LB agar + 1 mmol dm^−3^ GSH	4.3±0.6 *	0.0±0.0 *	0.0±0.0 *
LB agar + human serum 50% *v/v*	2.3±0.6 *	1.7±0.3 *	ND

The average zone of inhibition (mm) surrounding 1.25×1.25 cm dressing samples applied to bacterial lawns. ± SD, n = 3, * denotes a significant difference from LB agar only control (Student's *t*-test P<0.01). GSH, reduced glutathione; LB, Luria-Bertani; ND, Not determined (conditions do not support growth).

### Cytotoxicity of silver towards primary human fibroblasts

Fibroblasts within the dermal layer of the skin are one of the most important cell types involved in wound healing. This is therefore the cell line of choice for assessing cytotoxicity of silver in wound dressings and medical devices. Primary cells are directly acquired from donor tissue and have a limited lifespan in cell culture. These cells are therefore preferred for cytotoxicity studies as they more closely reflect host responses *in vitro* than immortalized cell lines that may have changed significantly during routine culture in the laboratory.

**Figure 4:**
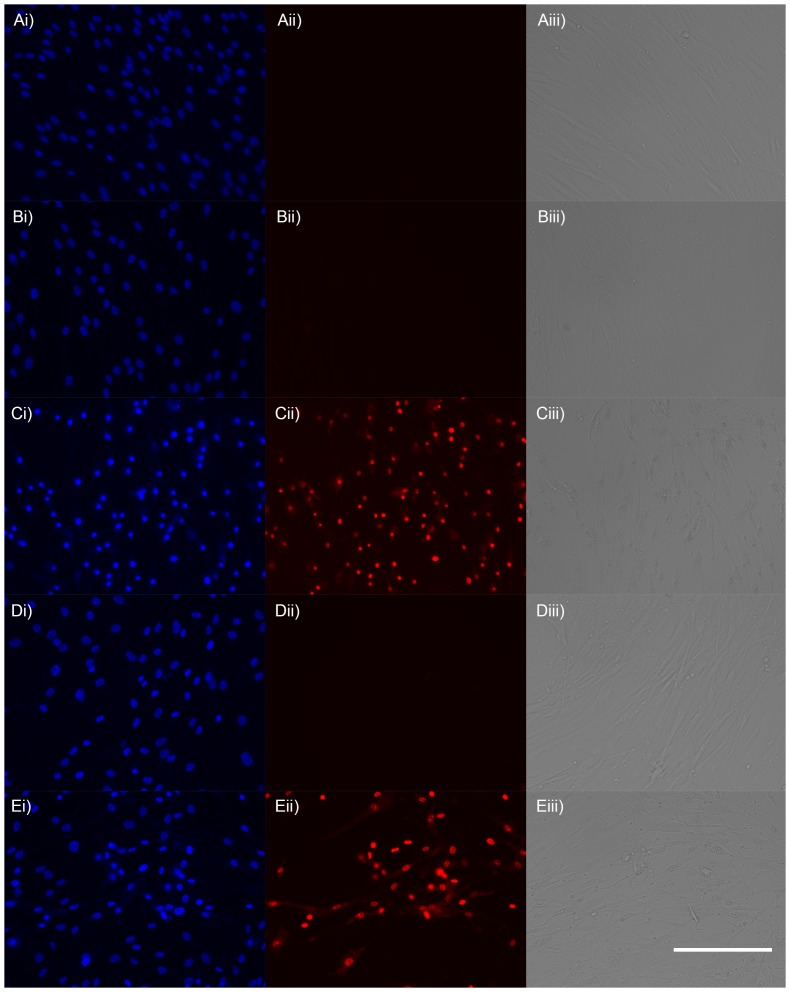
Micrographs of primary adult human dermal fibroblasts exposed to silver nitrate. Cells were exposed to AgNO_3_ at the indicated concentration for 24 hr: (A) 0 µmol dm^−3^ AgNO_3_; (B) 10 µmol dm^−3^ AgNO_3_; (C) 25 µmol dm^−3^ AgNO_3_; (D) 750 µmol dm^−3^ AgNO_3_ + 1 mmol dm^−3^ GSH; (E) 1 mmol dm^−3^ AgNO_3_ + 1 mmol dm^−3^ GSH. Images were captured for the same cells stained with i) NucBlue (Hoechst 33347), which stains all cell nuclei and ii) Propidium iodide, which stains nuclei of dead cells; (iii) Light microscope images show changes in cell morphology. GSH, reduced glutathione. Scale bar  =  200 µm

**Figure 5:**
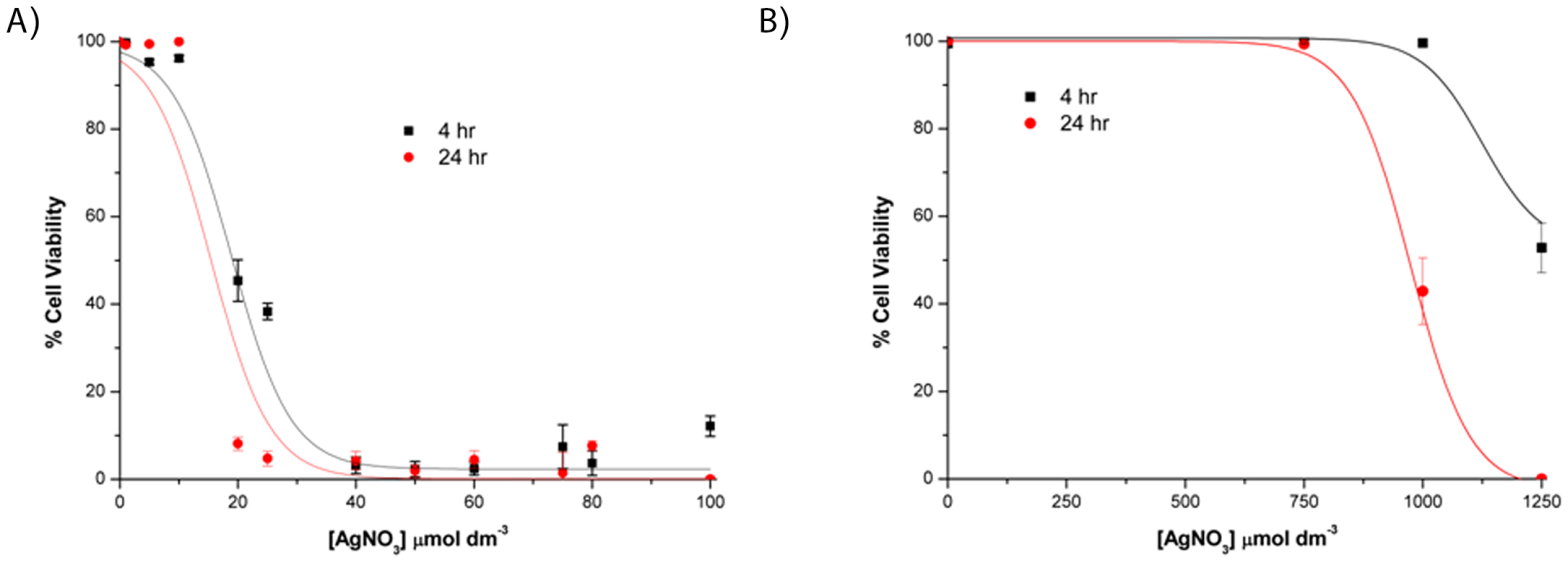
The cytotoxicity of silver nitrate to human skin cells. Viability of primary adult human dermal fibroblasts exposed to AgNO_3_ for 4 h or 24 h in (A) Medium 106; (B) Medium 106+1 mmol dm^−3^ GSH. Sigmoidal curves were fitted using the Boltzman function in OriginPro8 (OriginLab). Error bars  =  SEM, n = 4. GSH, reduced glutathione.

**Figure 6:**
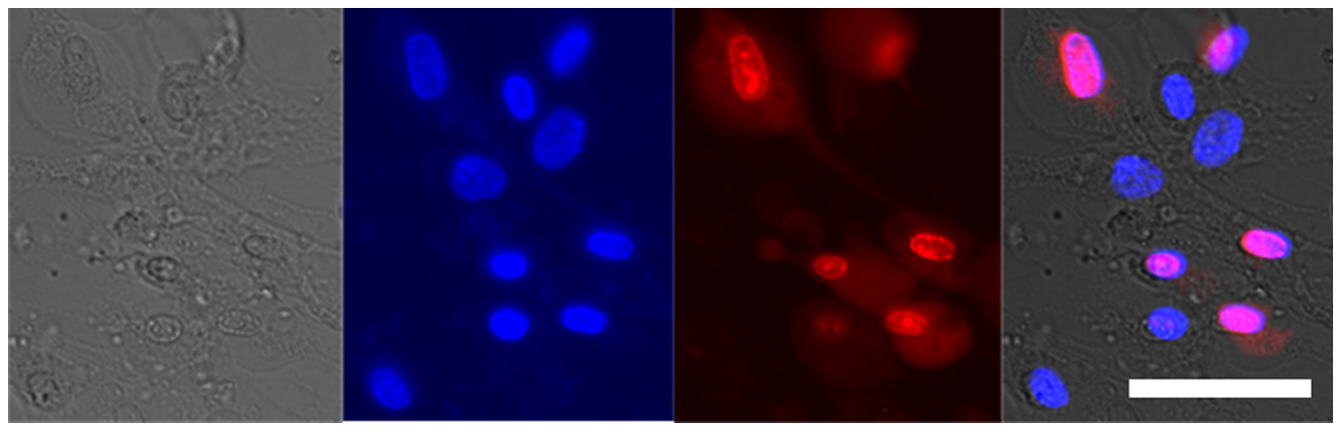
Nuclear condensation in human skin cells exposed to the minimum cytotoxic concentration of silver nitrate. Primary adult human dermal fibroblasts were exposed to 20 µmol dm^−3^ AgNO_3_ for 4 hr. Images were captured for the same cells: (A) Light microscope image shows cellular morphology; (B) stained with NucBlue (Hoechst 33347), which stains all cell nuclei; (C) stained with propidium iodide, which stains nuclei of dead cells (NB. areas of nuclear condensation are indicative of apoptosis); (D) Composite image of A, B and C. GSH, reduced glutathione. Scale bar  =  50 µm.

The cytotoxic concentration (CC_50_) of AgNO_3_ to primary human dermal fibroblasts was 23 µmol dm^−3^, which is in the same range as the MIC to the bacteria tested in this study (Table 1). Exposure of cells to 10 µmol dm^−3^ AgNO_3_ for 24 hours had no visible effect on cell morphology (cells remained elongated) or viability (cell nuclei stained with NucBlue, but not propidium iodide) and cells maintained a confluent, adherent monolayer (Figure 4B). In contrast, cells exposed to 25 µmol dm^−3^ AgNO_3_ were rounded (as opposed to elongate) and had begun to detach from the culture plate. The nuclei of the majority of these cells stained with propidium iodide indicating compromised cell membrane integrity (Fig 4Cii) equating to a 60% and 95% reduction in viability at 4 hr and 24 hr respectively (Figure 5A). Furthermore, the nuclei of the cells stained with propidium iodide showed signs of nuclear condensation, which is indicative of apoptosis or “programmed cell death” (Figure 6). The addition of 1 mmol dm^−3^ GSH to the cell culture medium increased the CC_50_ of AgNO_3_ to 982 µmol dm^−3^ after 24 hr (Table 1, Figure 5B). The addition of 1 mmol dm^−3^ sodium nitrate to the cell culture medium had no effect on cell morphology or viability relative to controls after 24 hr, indicating nitrate does not influence the cytotoxicity of AgNO_3_ (data not shown).

### Mechanism of thiol protection

To determine how extracellular R-SH reduce the toxicity of Ag^+^ we used ICP-OES to analyse the silver content of *S. aureus* exposed to 1 mmol dm^−3^ AgNO_3_ with and without the inclusion of an equimolar concentration of GSH in LB broth. Ag was detected in cells exposed to AgNO_3_ only at a concentration of 326±62 fg Ag/cell and 62.8±5.5 µg Ag/mg cell dry weight (± SEM, n = 4). In contrast, Ag was not detectable in cells exposed to LB only and AgNO_3_ + GSH. We therefore conclude that extracellular R-SH prevent Ag^+^ from binding to cells and this inactivates Ag^+^ toxicity.

## Discussion

Our findings prove that reduced thiol groups (R-SH) in the extracellular environment markedly reduce the antimicrobial efficacy and cytotoxicity of silver ions. When Ag^+^ and R-SH are added in a 1∶1 ratio the reaction of Ag^+^ with R-SH prevents Ag^+^ from interacting with cells thereby inactivating silver toxicity. GSH is the predominant low molecular weight thiol in humans, present in all cell types at a concentration of between 1 and 10 mmol dm^−3^ and in blood at approximately 1 mmol dm^−3^ [24], [25]. Our results show the addition of 1 mmol dm^−3^ GSH results in complete loss of antibacterial activity of Aquacel-Ag (Convatec) and Acticoat (Smith & Nephew) dressings to both *S. aureus* and *E. coli in vitro.* Given recent evidence that the toxicity of silver nanoparticles is dependent on the rate of dissolution of free Ag^+^ [6], [26], [27], extracellular R-SH will similarly reduce their antibacterial efficacy. The negative effect of complex-formation between biological R-SH groups and Ag^+^ should be considered in the future development of all novel silver coatings and nanoparticles. *In vitro* testing of silver-coated dressings and medical devices should be performed in biologically relevant media as the concentration of R-SH in standard bacterial culture media is typically much lower than in human blood and tissue. This is particularly relevant to the testing of sustained-release devices as the presence of R-SH in biological tissues could significantly affect the rate of dissolution of Ag^+^ and the duration of antimicrobial efficacy. Another consideration is that *in vitro* tests are typically performed in closed systems, which could exaggerate the longevity of antimicrobial action due to saturation of R-SH with Ag^+^. It seems likely that the constant replenishment of biological fluids containing R-SH would continue to limit the antimicrobial efficacy of Ag^+^ released from dressings/devices *in vivo*. Furthermore, the concentration and rate of Ag^+^ dissolution from antibacterial coatings on medical devices and wound dressings should be carefully controlled to minimize cytotoxicity towards dermal fibroblasts and other human cell types because this could reduce the rate of wound healing, as suggested elsewhere [28], [29]. Indeed, a recent Cochrane systematic review of the use of topical silver including silver sulphadiazine in the treatment of burns suggested that there is insufficient clinical evidence to support the hypothesis that such dressings do indeed promote healing or prevent infection [30].

Whilst it was not possible to use identical culture conditions for the bacterial and human cell assays in this study, we found that the toxicity of AgNO_3_ to both bacteria and human cells was within the same range, which is in agreement with the results of Greulich *et al*. [10]. The toxicity of silver is attributed to multiple factors including cell membrane damage, inhibition of respiratory enzymes, perturbation of metal ion homeostasis and generation of ROS that damage cellular components such as DNA and lipids. Several studies have demonstrated that the major target site(s) of Ag^+^ in Gram-negative bacteria are intracellular. Firstly, low-level silver resistance by adaptation of *E. coli* to increasing concentrations of AgNO_3_ was achieved by both decreased outer membrane permeability (due to a decrease in porin proteins that form membrane channels in the outer membrane) and active efflux of Ag^+^ from the cell [31]. Secondly, all known high-level silver resistance mechanisms in bacteria involve efflux pumps [32], [33]. Only one silver efflux system has been characterised at the molecular level to date and is encoded by the *sil* genes (*silRSE silCBA silP*) on the pMG101 plasmid of *Salmonella*. This system utilises a periplasmic Ag^+^-binding protein (SilE), which surprisingly lacks cysteine residues and instead coordinates 10 silver ions per polypeptide via 10 histidine residues [34]. The binding of silver ions to exposed thiol groups within a cell would have two complementary negative effects. Firstly, it might impair the functionality of any biomolecules to which it became bound and secondly it would reduce the cell's ability to neutralize natural ROS by depleting the effector molecules of the homeostatic antioxidant system such as GSH and cysteine. This would explain why silver ions often induce a measurable increase in intracellular ROS in both bacterial [35] and human cells [36]–[38], but do not directly generate ROS via Fenton-type reactions [35]. It should be noted that bacterial cells are much smaller than human cells and therefore contain less total GSH (or alternative low molecular weight thiols) per cell. Human cells also produce several forms of the cysteine-rich protein metallothionein (MT) that protect against oxidative damage and metal-ion toxicity [39], [40]. MT gene expression is induced by treatment with sub-inhibitory concentrations of silver, suggesting a role in cytoprotection against this specific stress [41], [42]. Furthermore, the majority of cytoplasmic proteins in a bacterium are maintained in the reduced state [16] and should therefore be more susceptible to Ag^+^ binding. With this in mind, it is surprising that bacterial cells are not much more sensitive to Ag^+^ than human cells. One possible explanation is that the most sensitive “targets” in bacteria and human cells lay in common essential biological processes or pathways. Xu *et al.* recently showed that silver specifically inhibits the activity of several dehydratases in *E. coli*, leading to destruction of the exposed 4Fe-4S clusters and the release of iron ions [43], which would generate intracellular ROS via Fenton-type reactions. In eukaryotic cells this would cause mitochondrial damage and trigger apoptosis, as observed in response to silver treatment [37], [42], [44], [45]. Whilst the exact mechanisms of silver toxicity are still unclear, this study has shown that extracellular thiols inactivate Ag^+^ toxicity to both prokaryotic and eukaryotic cells. By understanding the mechanisms of silver toxicity and the inactivation of this by thiols, it may be possible to design silver-based antibacterial coatings with improved efficacy and reduced cytotoxicity *in vivo*.

## Conclusions

In conclusion, we have demonstrated that biologically relevant compounds that contain reduced thiol groups such as GSH and cysteine, and other human blood components, significantly reduce the toxicity of silver ions to clinically relevant bacteria and human dermal fibroblasts (skin cells). These findings have important implications for the development and testing of novel antimicrobial coatings, particularly those intended for use in environments exposed to biological tissues or secretions such as wound dressings and indwelling medical devices.
